# HPV16 E6-specific T cell response and HLA-A alleles are related to the prognosis of patients with cervical cancer

**DOI:** 10.1186/s13027-021-00395-y

**Published:** 2021-09-16

**Authors:** Hongchao Cai, Yaning Feng, Peiwen Fan, Yuping Guo, Gulina Kuerban, Cheng Chang, Xuan Yao, Yanchun Peng, Ruozheng Wang

**Affiliations:** 1 The Third Affiliated Hospital of Xinjiang Medical University, Key Laboratory of Cancer Immunotherapy and Radiotherapy, Chinese Academy of Medical Sciences, Urumqi, China; 2 Key Laboratory of Oncology of Xinjiang Uyghur Autonomous Region, Urumqi, China; 3 State Key Laboratory of Pathogenesis, Prevention and Treatment of High Incidence Diseases in Central Asia, Urumqi, China; 4 Chinese Academy of Medical Sciences Oxford Institute (CAMS Oxford Institute), Oxford, UK

**Keywords:** Human papillomavirus 16, Cervical cancer, T cell immune response, E6 and E7 peptides, HLA

## Abstract

**Background:**

T cell epitopes are polypeptide fragments presented to T cell receptors by MHC molecules encoded by human leukocyte antigen (HLA) genes after antigen-presenting cell processing, which is the basis for the study of antigen immune mechanism and multi-epitope vaccine. This study investigated T cell response to HPV16 E6 and E7 in patients with cervical squamous cell carcinoma (CSCC). Also, the HLA-A allele distribution was compared among patients and evaluated as a factor to predict prognosis in these patients.

**Materials and methods:**

This study recruited a total of 76 patients with International Federation of Gynaecology and Obstetrics (FIGO) stage IIB–IIIB CSCC. Mononuclear cells were isolated from the peripheral blood before any treatment and then enzyme-linked immunosorbent spot (ELISpot) assay was employed to measure the E6 and E7-specific T cell response. HLA‐A alleles were typed using Sanger sequencing‐based typing techniques with DNA extracted from the peripheral blood. The correlation between the T cell responses, HLA‐A allele distribution and patient prognosis were analysed using the Kaplan–Meier method, univariate and multivariate Cox proportional hazard models.

**Results:**

The frequency of HPV E6-specific T cell responses in patients with pelvic lymph node metastasis was lower than that in patients without metastasis (*P* = 0.022). The 5-year overall survival (OS) rates of patients were 87.5% for those responding to multiple overlapping peptides, 72.7% for those responding to 1–2 overlapping peptides and 47.7% for non-responders (*P* = 0.032). Cox regression analysis indicated that the presence of HLA*A02:07 was independently associated with worse OS (hazard ratio [HR] 3.042; 95% confidence interval [CI] 1.348–6.862; *P* = 0.007), while concurrent chemoradiation therapy (CCRT) was independently associated with better OS (HR 0.475; 95% CI 0.232–0.975; *P* = 0.042).

**Conclusion:**

The results of our study demonstrated that the level of HPV16 E6-specific T cell response and HLA*A02:07 were correlated with prognosis in patients with advanced CSCC.

**Supplementary Information:**

The online version contains supplementary material available at 10.1186/s13027-021-00395-y.

## Introduction

Cervical cancer (CC) is one of the most frequent malignant diseases among women worldwide, with around 570,000 cases diagnosed and 311,000 deaths registered in 2018 [1]. Persistent high-risk human papillomavirus (hr-HPV) infection primarily contributes to the initiation and development of CC. hr-HPV strains 16 and 18 are predominantly present in 70% of CC cases, while other hr-HPV strains cause an additional 10% of cases [2]. Although vaccination programs and screening by HPV tests may decrease the future occurrence of CC [3], diagnosed patients still rely on traditional treatment to control the disease progression, while the prognosis remains poor for patients with locally advanced disease [4]. The KEYNOTE-028 and CHECKMATE 358 trials showed objective remission rates (ORRs) for CC immunotherapy as a second-line treatment of only 17% and 26.3%, respectively [5, 6]. Therefore, it is still worthwhile to develop treatment such as therapeutic vaccine that can benefit patients who do not respond well to the current treatment modality.

The tumour immune surveillance in human body depends mainly on T-cell-mediated immunity [7]. The HPV-specific T cell immune response plays a key role in clearing HPV infection and controlling HPV-related diseases by initiating the immune response mediated by CD8 + and CD4 + T cells to eliminate the host cells having malignant transformation [8]. Although prophylactic HPV vaccines with high efficiency and safety have been approved and widely used, they cannot eliminate the existing HPV infection. The HPV genome contains eight open reading frames (ORFs) encoding eight proteins. Among them, proteins E6 and E7 are essential drivers of carcinogenic transformation [9], making them ideal targets for the clearance of HPV-related precancerous lesions and cancers. E7 was the first oncogene identified among all HPV oncogenes and has been proved to impair the function of tumour suppressor retinoblastoma protein pRB [10]. E6, on the other hand, encodes protein which can hinder apoptosis by inducing tumour suppressor p53 degradation [11]. A variety of HPV therapeutic vaccines based on peptides, DNA, and proteins are being developed. The utility of therapeutic vaccines based on E7 has been demonstrated in the treatment of cervical precancerous lesions. Among the DNA-based vaccine, VGX-3100, for which a phase 2b trial indicated that 49.5% and 30.6% of patients treated and subjects of the placebo group showed histopathological regression to low grade squamous intraepithelial lesion (LSIL) or normal pathology at 36 weeks(*p* = 0.034) [12]. Kim et al. synthesised overlapping peptides E7_50-59_ (AHYNIVTFCC) and E7_52-61_ (YNIVTFCCKC) and confirmed sensitization of CD8 + cytotoxic T lymphocytes (CTLs) by these peptides as an antitumor effect against CC cells [13]. However, there is still a lack of clinical evidence on which epitopes from E6 and E7 can elicit the most robust immune response in cervical squamous cell carcinoma (CSCC) patients. The identification of T cell epitopes is a vital step in the design of vaccine and immunotherapy for cervical cancer.

Human leukocyte antigen (HLA) is a vital part of T‐cell immunity and shows genetic heterogeneity among populations. HLA families are classified as HLA-I, -II, and III and exhibit codominant alleles comprising combinations of gene sequences generated by mutation, conversion, and recombination [14]. The heterogeneity of HLA is the molecular biological factor for distinct immune responses. Six major genes (HLA-A, HLA-B, HLA-C, HLA-D, HLA-E, HLA-F) are encoded by major histocompatibility complex (MHC)-I. These genes encode a set of proteins that present epitopes to specific receptors on T cells. Specifically, HLA-I and HLA-II molecules expressed on cell surface bind to HPV antigenic peptides and form antigenic peptide-MHC class I and II complexes [15]. The target molecules are presented to CD8 + and CD4 + T cells to initiate an immune response, activate CTLs and kill tumour cells [16]. Whole-exome sequencing confirmed somatic mutations in 8% of HLA-A and 6% of HLA-B genes exclusively in CSCC patients [17]. HLA heterogeneity contributes to the different response to the peptide-based vaccine due to their varied ability to present epitopes, which is partly reflected in different HLA allele distribution in CC patient compared to the general population. For instance, Madeleine MM et al. reported that alleles A*03:01 were significantly associated with an elevated risk of CC [18], while another study reported the association between the B*07:02 allele and an increased predisposition to cervical adenocarcinoma [19].

It is worthwhile to identify more epitopes as a target to design T cell-based therapeutic interventions that can improve the curative effect of patients with cervical cancer who the current treatment can not sufficiently control. Our study assessed the E6 or E7-specific T cell responses in peripheral blood mononuclear cells (PBMCs) from patients with FIGO stage IIB–IIIB CSCC, analysed the relationship between HLA-A allele distribution and prognosis. We hypothesised that the number of E6 an E7 epitopes to which T cells can respond in one patient was related to the prognosis of patients. We also evaluated HLA-A allele distribution as prognostic factor among the clinical characteristics so as to provide clinical evidence for the design of therapeutic interventions.

## Materials and methods

### Subjects

This study included 76 patients with unresectable CSCC who were admitted to the Affiliated Tumour Hospital of Xinjiang Medical University (Urumqi, China) between June 2013 and September 2015. The median age of the patients was 55 years (range 34–85 years). The inclusion criteria were: (1) diagnosis of primary squamous cell carcinoma (SCC) that was clinically classified as stage IIB–IIIB according to the FIGO classification; (2) no infectious diseases or autoimmune diseases such as hepatitis and syphilis; and (3) Karnofsky Performance Status Scale scores of ≥ 70 points. Patients who failed to complete radical radiotherapy (RT) were excluded. Patients were staged according to 2009 the FIGO recommendations. Written informed consent was obtained from all patients enrolled. Ethical approval was obtained from the Affiliated Tumour Hospital of Xinjiang Medical University.

### Interferon-γ ELISpot assay

PBMCs were isolated from fresh heparinized blood by Ficoll-Hypaque density gradient centrifugation. HPV16 E6 and E7-specific T cell responses were determined with interferon-γ ELISpot assay. Specifically, we referred to the GenBank (https://www.ncbi.nlm.nih.gov/genbank/) database and design 18 and 11 overlapping peptides representing the complete sequence of HPV16 E6 and E7, respectively (Tables 2 and 3). The 18-mer peptides were synthesised from Sigma (Sigma‐Aldrich, Saint Louis, USA) with 10 amino acid overlapping between the adjacent peptides. A total of 0.2 million PBMCs were stimulated with each of the single overlapping peptides as described previously in a polyvinylidene fluoride (PVDF) plate (Millipore, Bedford, USA) coated with antibodies from Human IFN-gamma ELISpot BASIC kit (ALP) (Mabtech, Australia). The positive and negative controls were PBMCs stimulated by 20 μg/mL phytohemagglutinin and unstimulated PBMCs, respectively. After being incubated overnight in 5% CO2 at 37 ℃, the ELISpot plate was developed according to the instruction from the manufacturer (Mabtech, Australia). AID EliSpot Reader was used to read and count spot-forming cells (SFCs). The peptide-specific T cell response was considered positive when more than 25 SFCs were detected in the peptide-treated well, as well as three-fold more SFCs than the negative controls [20].

### HLA-A typing

Genomic DNA was extracted using the blood genomic DNA extraction kit (Tiangen, Beijing, China) from patients’ peripheral blood. The DNA concentration and purity were determined with the DU-640 UV spectrophotometer (Thermo Fisher, Waltham, USA). The DNA had an A260/A280 between 1.8 and 2.0 and the final concentration was adjusted to 0.3–0.5 μg/μL. The HLA‐As were typed using Sanger sequencing‐based method.

### Clinical treatment

The patients were treated according to the 2013 National Comprehensive Cancer Network (NCCN) Cervical Cancer Guidelines. Radical radiotherapy (RT) or Concurrent chemoradiation therapy (CCRT) was administered to all patients in our study. The main RT techniques were high-dose-rate brachytherapy, three-dimensional conformal radiation therapy, or intensity‐modulated radiation therapy and were selected according to the patient conditions, with 50–50.4 Gy whole-pelvic external beam with 30 Gy high-dose-rate brachytherapy recommended. The treatment for pelvic lymph node (PLN) metastasis consisted in the synchronous integration increment technique and an additional 10–15 Gy. Due to geographical restrictions and intolerance to chemotherapy regimens, only 60% (46/76) of patients received simultaneous chemotherapy during RT. TP (taxol 135 mg/m^2^ + 50 mg/m^2^ cisplatin) or DDP (30 mg/m^2^ cisplatin) per week were administered for a total of four cycles.

### Follow-up

The median follow-up time of the censored cases was 58.4 months from the date of diagnosis. In the first 2 years, follow-up was conducted every 3 months after treatment, and then once every 6 months. Overall survival (OS) was defined as the interval from the initial diagnosis to death or last follow-up.

### Statistical analysis

Statistical analyses were performed using IBM SPSS Statistics for Windows, version 25.0. Graphs of correlation analysis were generated using GraphPad Prism 8. The characteristics of patient tumours and the distributions of HLA‐A alleles were compared using chi-square test. Differences were considered statistically significant for *P* < 0.05. The Kaplan–Meier estimate was used to perform survival analysis. We performed univariate and multivariate Cox regression analyses to assess the prognostic factors of patients.

## Results

### HPV 16 E6-specific T cell response was related to pelvic lymph node metastasis

Patients with stage 2 or 3 disease comprised 59.2% (45/76) and 40.8% (31/76) of the study population, respectively (Table1). Thirty-nine patients (51.3%) had PLN metastasis at the time of diagnosis. The frequency of HPV16 E6 peptide-specific T cell response in patients with PLN metastasis was significantly lower than in those who had no metastasis (*P* = 0.022) (Table 1). However, the frequencies of E7 peptide-specific T cell responses between patients with or without PLN metastasis did not differ significantly (Table 1). The E6 or E7 peptide-specific T cell response frequency also did not show differences when the patients were grouped according to age, clinical stage, tumour size, tumour type, histologic grade, HPV 16 status, or levels of carcinoembryonic antigen (CEA), tumour-specific growth factor (TSGF) or squamous cell carcinoma antigen (SCC-Ag).

**Table 1:** The relationship between frequency of HPV16 E6 or E7-specific T cell response in peripheral blood and clinical features in CSCC patients

Factor	N	E6-peptide specific T cell response		E7-peptide specific T cell response	
		Frequency (%)	*P*-value	Frequency (%)	*P*-value
Age (years)					
≤ 55	38	52.63	0.105	31.58	0.807
> 55	38	34.21		34.21	
Stage					
IIb	45	37.78	0.232	35.56	0.552
IIIa-b	31	51.61		29.03	
Tumor size (cm)					
< 5	40	37.50	0.272	35.00	0.681
≥ 5	36	50.00		30.56	
Tumor type					
Cauliflower	33	45.45	0.859	27.27	0.338
Hollow	6	33.33		16.67	
Nodular	37	43.24		40.54	
Histologic grade					
Poor	18	38.89	0.310	27.78	0.163
Moderate	46	45.65		34.78	
Well	2	100		100	
Papillary	10	33		20.00	
HPV 16					
Negative	14	50.00	0.582	42.86	0.380
Positive	62	41.93		30.64	
TSGF					
Normal	45	44.45	0.828	37.78	0.275
Abnormal	31	41.94		25.81	
CEA					
Normal	59	42.37	0.731	33.90	0.729
Abnormal	17	47.06		29.41	
SCC					
Normal	20	50.00	0.489	30.00	0.748
Abnormal	56	41.07		33.93	
Pelvic lymph nodes					
Negative	37	56.76	0.022	40.54	0.167
Positive	39	30.77		25.64	

HPV, human papillomavirus; TSGF, tumour-specific growth factor; CEA, carcinoembryonic antigen; SCC, squamous cell carcinoma antigen

### T cell response to multiple E6 or E7 peptides can be found in some CSCC patients

**Fig. 1:**
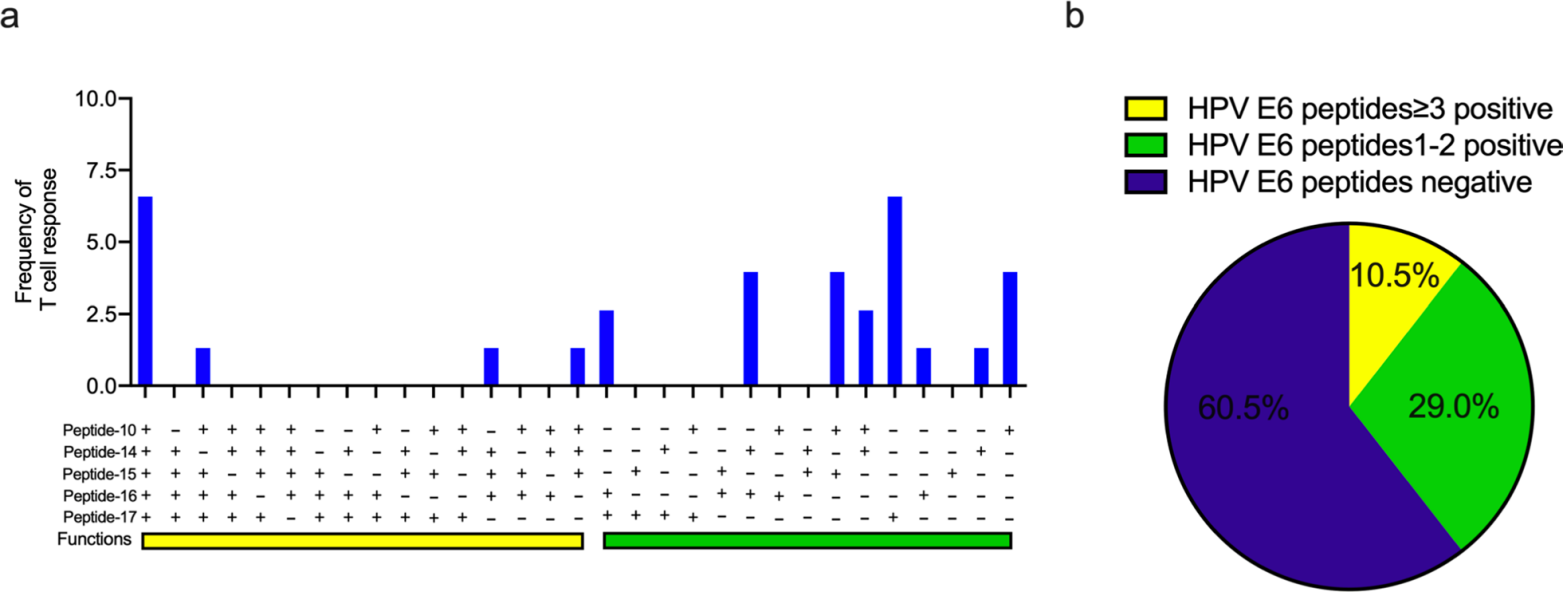
HPV 16 E6-specific T cell response in peripheral blood from CSCC patients. Among the 18 overlapping peptides of E6, 5 peptides which most frequently stimulated T cell response in the CSCC patients were identified (**a**). **b** Shows the percentage of patients responding to single, 1–2 or more than 2 peptides of HPV16 E6

To see if there existed dominant peptides which were responsible for the T cell response in the CSCC patient, we compared and summarised the frequencies and intensities of T cell response to the single E6 and E7 peptides. Of the 18 peptides representing E6, peptide-10 CLKFYSKISEYRHYCYSV_73-90_ and Peptide-14 DLLIRCINCQKPLCPEEK_105-122_ could most frequently elicit T cell response in all of the patients (19.74%), while peptide-15 CQKPLCPEEKQRHLDKKQ_113-130_ showed the highest intensity among those who responded (49.82 SFC/10^6^) (Table 2). In addition to these two peptides, T cell response to E6-16 and 17 peptides were also frequent in the 76 patients (Table 2). Then we analysed the cumulative frequencies of T cell response to one or multiple of the 5 E6 peptides. We found that 10.5% (8/76) of patients had T cell responses to over three of the five E6 peptides, while 29.0% (22/76) responded to one or two of the five peptides (Fig. 1a, b). The details of the E6 peptide-specific immune response are listed in Additional file 1: Table S1.

**Table 2:** The frequency and intensity of HPV16 E6 single peptide-specific T cell response

HPV16 E6 peptide	Squamous cell carcinoma of cervix (n = 76)	
	Frequency (%)	Mean magnitude (SFC/10^6^)
E6 Peptide-1MHQKRTAMFQDPQERPRK_1-18_	6.58	39.80
E6 Peptide-2 FQDPQERPRKLPQLCTEL_9-26_	9.21	44.00
E6 Peptide-3 RKLPQLCTELQTTIHDII_17-34_	9.21	41.00
E6 Peptide-4 ELQTTIHDIILECVYCKQ_25-42_	10.53	41.62
E6 Peptide-5 IILECVYCKQQLLRREVY_33-50_	10.53	40.37
E6 Peptide-6 KQQLLRREVYDFAFRDLC_41-58_	6.58	45.20
E6 Peptide-7 VYDFAFRDLCIVYRDGNP_49-66_	9.21	45.85
E6 Peptide-8 LCIVYRDGNPYAVCDKCL_57-74_	9.21	46.14
E6 Peptide-9 NPYAVCDKCLKFYSKISE_65-82_	10.53	38.37
E6 Peptide-10 CLKFYSKISEYRHYCYSV_73-90_	19.74	40.20
E6 Peptide-11 SEYRHYCYSVYGTTLEQQ_81-98_	11.84	44.33
E6 Peptide-12 SVYGTTLEQQYNKPLCDL_89-106_	7.89	45.50
E6 Peptide-13 QQYNKPLCDLLIRCINCQ_97-114_	10.53	33.50
E6 Peptide-14 DLLIRCINCQKPLCPEEK_105-122_	19.74	44.20
E6 Peptide-15 CQKPLCPEEKQRHLDKKQ_113-130_	14.47	49.82
E6 Peptide-16 EKQRHLDKKQRFHNIRGR_121-138_	17.11	37.3
E6 Peptide-17 KQRFHNIRGRWTGRCMS_129-146_	17.11	47.84
E6 Peptide-18 GRWTGRCMSCCRSSRTRR_137-154_	10.53	44.37

HPV, human papillomavirus

**Fig. 2:**
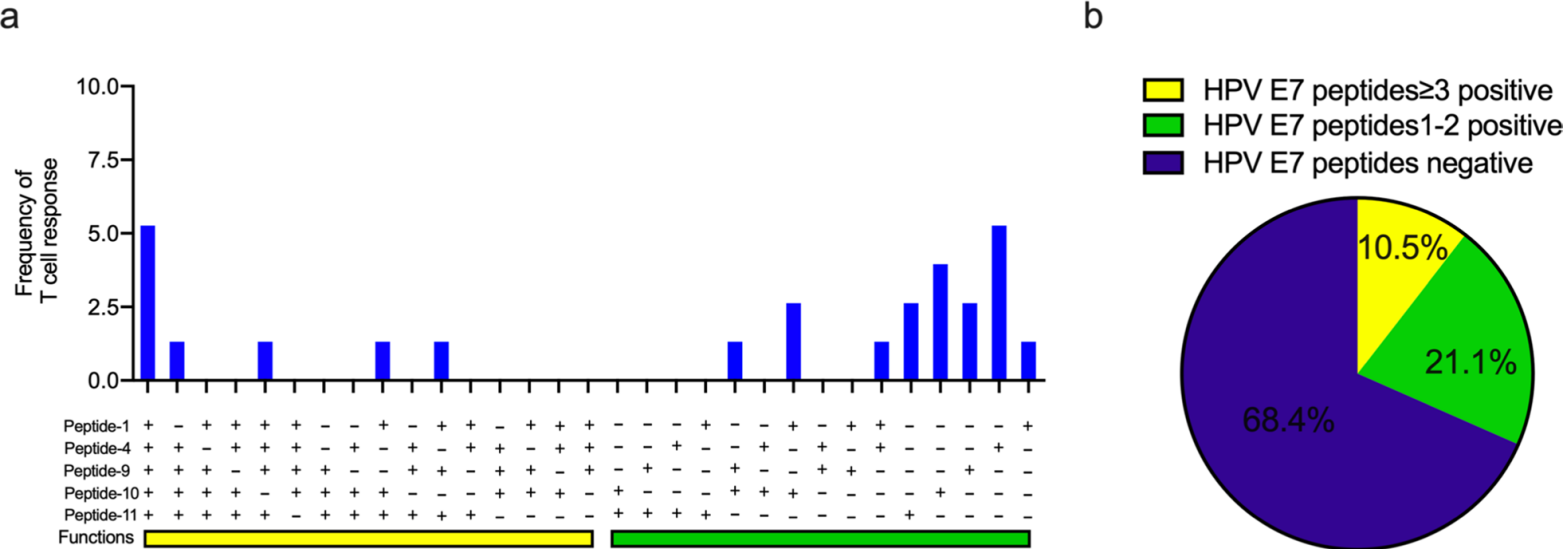
HPV 16 E7-specific T cell response in peripheral blood from CSCC patients among the 11 overlapping peptides of E7, 5 peptides which most frequently stimulated T cell response in the CSCC patients were identified (**a**). **b** Shows the percentage of patients responding to single, 1–2 or more than 2 peptides of HPV16 E7

As for the E7 protein, T cell response to E7-10 HVDIRTLEDLLMGTLGIV_73-90_ demonstrated the highest frequency and intensity (Table 3), with frequency followed by E7-1 MHGDTPTLHEYMLDLQPE_1-18_, E7-4 YEQLNDSSEEEDEIDGPA_25-42_, E7-9 LRLCVQSTHVDIRTLEDL_65-82_, and E7-11 DLLMGTLGIVCPICSQKP_81-98_. The frequencies of T-cell response stimulated by over three or 1–2 E7 peptides were 10.5% (8/76) and 21% (16/76), respectively (Fig. 2a, b). The details of the E7 peptide-specific immune response are listed in Additional file 1: Table S2.

**Table 3:** The frequency and intensity of HPV16 E7 single peptide-specific T cell response

HPV16 E7 peptide	Squamous cell carcinoma of cervix (n = 76)	
	Frequency (%)	Mean magnitude (SFC/10^6^)
E7 Peptide-1 MHGDTPTLHEYMLDLQPE_1-18_	15.79	46.92
E7 Peptide-2 HEYMLDLQPETTDLYCYE_9-26_	11.84	39.78
E7 Peptide-3 PETTDLYCYEQLNDSSEE_17-34_	10.53	45.37
E7 Peptide-4 YEQLNDSSEEEDEIDGPA_25-42_	15.79	52.00
E7 Peptide-5 EEEDEIDGPAGQAEPDRA_33-50_	11.84	44.00
E7 Peptide-6 PAGQAEPDRAHYNIVTFC_41-58_	9.21	43.43
E7 Peptide-7 RAHYNIVTFCCKCDSTLR_49-66_	11.84	40.89
E7 Peptide-8 FCCKCDSTLRLCVQSTHV_57-74_	11.84	46.33
E7 Peptide-9 LRLCVQSTHVDIRTLEDL_65-82_	13.16	53.10
E7 Peptide-10 HVDIRTLEDLLMGTLGIV_73-90_	15.79	55.33
E7 Peptide-11 DLLMGTLGIVCPICSQKP_81-98_	15.79	46.83

HPV, human papillomavirus

### HPV16 E6 overlapping peptide-specific T cell immune responses predicted the OS of patients with CSCC

**Fig. 3:**
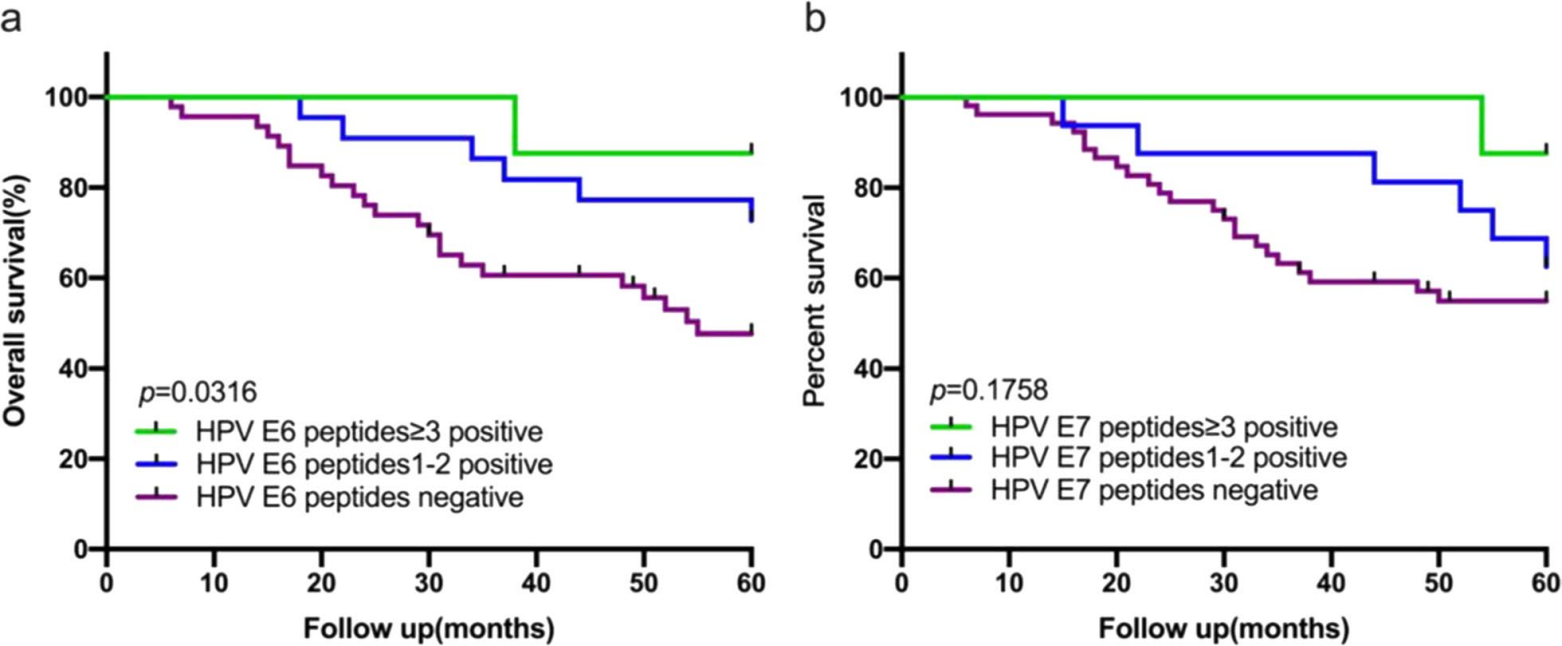
The relationship between the number of E6 or E7 peptides inducing T cell response and the prognosis of CSCC. **a** Kaplan–Meier plot for overall survival (OS) analysis of CSCC patients with different number of HPV E6 peptides-specific T cell response in five most frequently peptides. **b** Kaplan–Meier plot for overall survival (OS) analysis of CSCC patients with different number of HPV E7 peptides-specific T cell response in five most frequently peptides

The correlation between HPV 16-specific T cell response and prognosis in CC patient can provide evidence to the design of therapeutic intervention. The Kaplan–Meier plots showed the impact of different peptide-specific T cell responses to the five most frequently peptides on OS (Fig. 3). The 5-year OS rates of patients with E6-specific T cell response to at least three peptides was 87.5%, compared to 72.7% in those who responded to only 1–2 peptides. But both groups showed significantly higher OS rates than those who did not respond to the E6 peptides (47.7%) (*P* = 0.0316) (Fig. 3a). No such differences were observed in the E7-specific T cells response, with 5-year OS rates of the multi-, 1–2, and 0-peptide responders 87.5%, 62.5%, and 54.9%, respectively (*P* = 0.1758) (Fig. 3b).

### HLA‐A allele was related to the quantity of E6 peptides eliciting T cell response in the patient

**Fig. 4:**
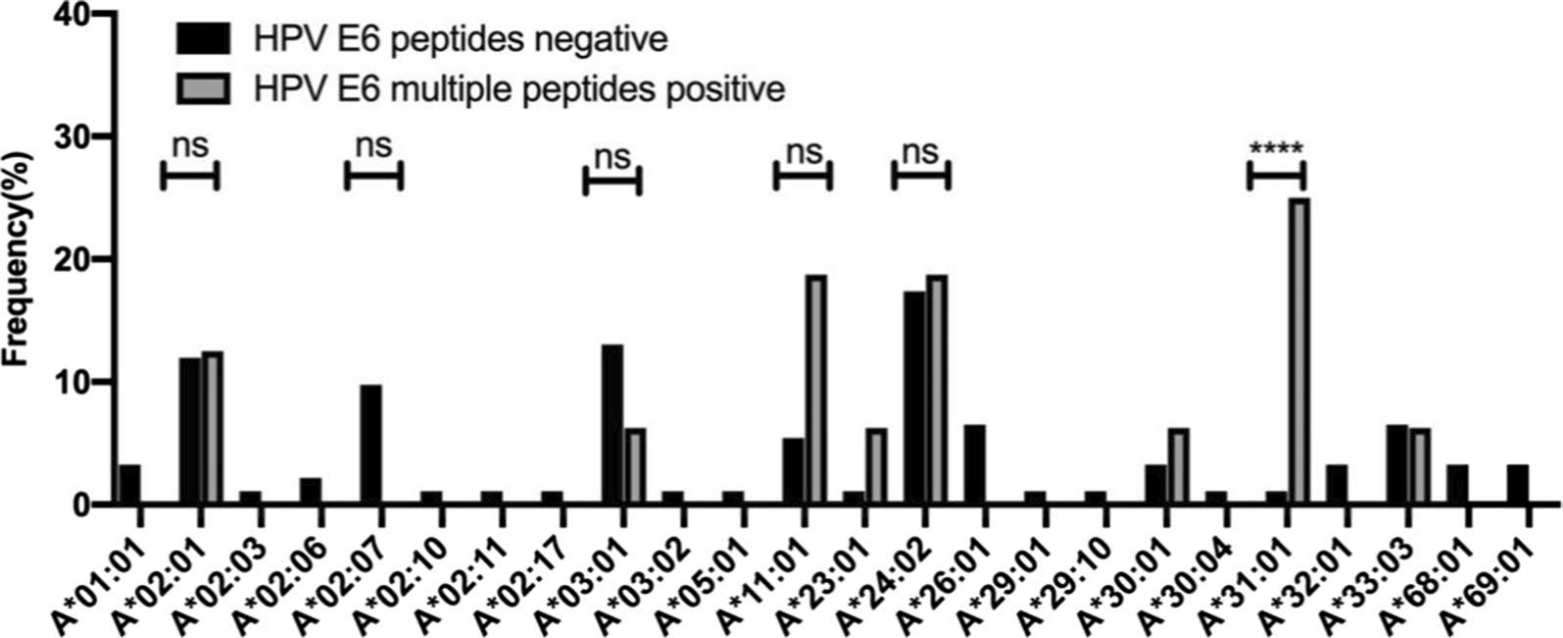
The HLA-A allele distribution in patients with multiple-E6 peptide (n = 8) or no T cell response (n = 46)

Of particular importance is the CD8 T cell response to eliminate transformed cells infected by HPV. MHC I molecules encoded by class I HLA genes are responsible for antigens presentation to CD8 T cells. Association of HLA-A alleles, one of the locus encoding MHC I molecules, to cervical cancer prognosis was reported. We then looked into the question if the impact of HLA-A to disease outcome was related to the quantities of peptides inducing T cells response per patient. As shown in Fig. 4, the HLA‐A allele distributions differed between the group with multiple E6-peptide response and the group with no E6 response at all. Of the 24 alleles genotyped, patients with multiple E6 peptide-specific T cell responses were more significantly frequent to be HLA‐A*31:01 compared to the patients showing no response to E6 peptides (25% vs. 1.09%, *P* < 0.0001). The frequency of allele A*02:07 in the group with no E6 response was 9.78% (9/92) while this allele was not found in patients possessing multiple E6 peptide-specific T cell response. However, there was no such difference between the two groups in common alleles, including A*02:01, A*11:01, and A*24:02.

### Prognosis evaluation with HLA-A alleles and E6-specific T-cell response in patients with CSCC

**Fig. 5:**
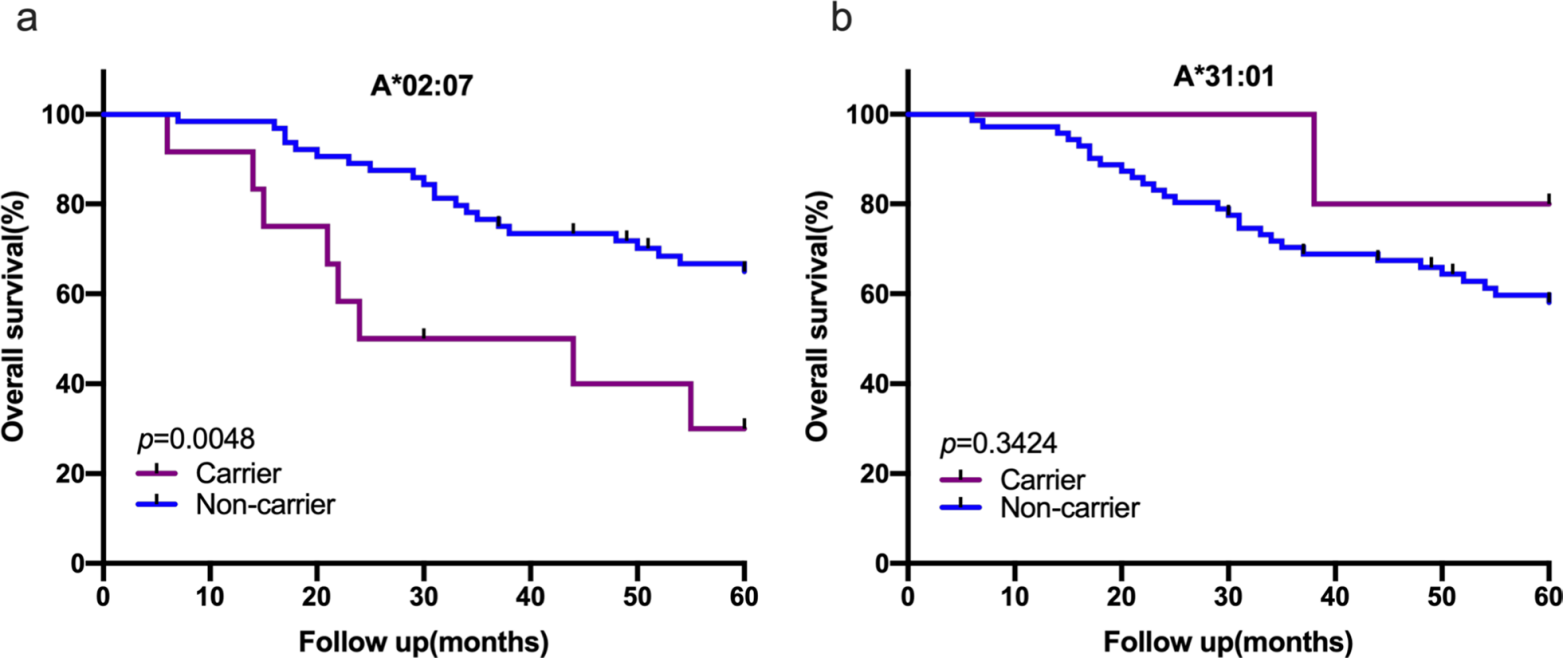
HLA-A alleles are related to OS in patients with CSCC. **a** Kaplan–Meier curves HLA-A02:07 carrier and non-carrier (log-rank test, *P* = 0.005). The patients of HLA-A*02:07 carrier displayed poor OS compared to non-carrier patients. **b** Kaplan–Meier curves HLA-A*31:01 carrier and non-carrier (log-rank test, *P* = 0.342)

We investigated the value of HLA-A alleles and E6-specific T cells response as prognostic factors in patients with CSCC. The 5-year OS rates of A*02:07 carriers and non-carriers were 30% and 64.97%, respectively (Fig. 5a). Eight of the 76 patients with A*02:07 died of CSCC. A*02:07 carriers showed a significant lower OS (*P* = 0.005). Meanwhile, one of the five patients with A*31:01 allele died of CSCC. However, A*31:01 did not affect OS (*P* = 0.342) (Fig. 5b).

### Prognostic factors of OS in patients with CSCC

Univariate analyses (Table 4) demonstrated that PLN metastasis (hazard ratio [HR] 2.186; 95% confidence interval [CI] 1.037–4.605; *P* = 0.021) and HLA A*02:07 (HR 3.042; 95% CI 1.348–6.862; *P* = 0.007) were significant risk factors related to a poor prognosis. Patients who received CCRT (HR 0.475; 95% CI 0.232–0.975; *P* = 0.042) had a better prognosis. In multivariate analysis, CCRT (HR 0.425; 95% CI 0.475 (0.205–0.881); *P* = 0.021) and HLA A*02:07 (HR 2.914; 95% CI 0.475 (1.270–6.686); *P* = 0.012) were independently associated with OS.

**Table 4:** Cox regression analyses of overall survival in CSCC patients

Factors	OS (univariate analysis)		OS (multivariate analysis)	
	HR (95% CI)	*P*-value	HR (95% CI)	*P*-value
Age	1.755 (0.845–3.645)	0.131	–	–
Stage	1.281 (0.625–2.625)	0.499	–	–
Tumor size	1.307 (0.637–2.680)	0.466	–	–
Tumor type	1.279 (0.872–1.877)	0.208	–	–
Histologic grade	0.863 (0.572–1.302)	0.482	–	–
HPV	1.509 (0.671–3.394)	0.320	–	–
Pelvic lymph nodes	2.186 (1.037–4.605)	0.040	2.053 (0.958–4.397)	0.064
Therapy	0.475 (0.232–0.975)	0.042	0.425 (0.205–0.881)	0.021
Multiple overlapping-peptide response	0.227 (0.031–1.666)	0.145	0.307 (0.040–2.352)	0.256
HLA A*02:07	3.042 (1.348–6.862)	0.007	2.914 (1.270–6.686)	0.012

HPV, human papillomavirus; HLA, human leukocyte antigen

## Discussion

Recent advances have been achieved in the research and development of therapeutic HPV vaccines to enhance T cell activity, including protein, polypeptide and live vector, nucleic acid, and cellular vaccines. Youn JW et al. reported that GX-188E vaccination enhanced the proportion of clinical responders to immune checkpoint inhibitors (ICBs) by adding HPV-specific T cells [21]. Compared with pembrolizumab alone, the ORR increased from 17 to 42% [5, 21]. Another study also confirmed the necessity for therapeutic vaccine-induced T cell populations in immunotherapy. The overlapping long peptide vaccine ISA101, which is no longer restricted by HLA, covers the complete HPV16 E6 and E7 oncoprotein sequences. The ORR of ISA101 combined with Anti-PD-1 therapy in patients with incurable HPV16-positive malignant neoplasms was 33% [22]. While these vaccines have great therapeutic promise, they are still in the stage of basic research and clinical trials. One of the key questions remains which epitopes to choose so as to trigger the optimal immune response against the tumour and metastasis. It is well known that the expression peaks of HPV16 E6 and E7 in HPV infected cells have been established and transformed. Thus, in our study, we tried to uncover the clinical relevance of E6 and E7-specific T cell response in CSCC patients. We used overlapping peptides of HPV 16 E6 and E7 to determine the antigen-specific T cell response, and investigated the different HLA-A distribution in patients with CSCC. Meanwhile, combined with clinical characteristics, prognostic factors in patients with CSCC were analysed.

FIGO 2018 updated the staging system for CC to indicate that cases with PLN status are categorized as stage IIIC1 [23]. In our study, both E6 and E7-specific T cell response can be observed in some CSCC patients. Of note, we found that patients without PLN metastasis were more likely to exhibit E6-specific T cell responses, which indicates E6-specific T cells response could contribute to the inhibition of metastasis, which was not seen with E7-specific T cell response. Therefore, HPV-specific T cell response contributes to inhibiting tumour development and is potentially related to a better prognosis. In addition, it is noted that the frequency of T cell response in HPV-16 negative patients is higher than that in HPV-16 positive patients. We consider there are two possible reasons. As we have collected before treated blood samples, HPV-positive patients may have a certain degree of T cell function exhaustion after a period of the antigen-specific immune response. At this time, antigen stimulation may affect the positive response rate. In addition, the possible bias is due to the relatively more minor number of HPV-negative patients.

Epitope prediction is essential for the design and development therapeutic vaccines against HPV-related diseases. Compared with E6 peptides, E7 peptides are more frequently used in current research of peptide vaccines for HPV-induced CC [24–26] as the E7 protein is smaller with fewer amino acids thus more convenient for screening. However, vaccines targeting E7 might not be able to elicit T cell response indiscriminately in all CC patients. Chenzhang Y et al. identified E7 YMLDLQPETTDL_11-22_ as crucial regions comprising the majority of predicted antitumor epitopes [27]. Our study shows that the overlapping peptide of E7 peptide-1 and E7 peptide-2 covered the E7 YMLDLQPETTDL_11-22_. But they do not dominate in the specific immune response. Although the positive response rate of E7 peptide 1 was higher than peptide 2. Besides, we also found similar results to our study. Ressing ME et al. showed that E7-derived peptides (YMLDLQPETT_11-20_, LLMGTLGIV_82-90_, TLGIVCPI_86-93_) may represent naturally processed human CTL epitopes of HPVI6 and have more consistent with our E7-specific T cell response [28]. An early study confirmed that the immune response of CD8 + T cells to HPV-16 E6 antigens rather than E7 antigens was associated with better clinical outcomes [29]. In our study, we found that the patients responding to more than two of the five top-responsive peptides were more likely to have better prognosis compared to those who exhibited no E6 response. This kind of correlation was not seen with E7 peptide. Our study demonstrate that E6-specific T cell response is more relevant to the clinical outcome of the CC, potentially due to a broader epitope repertoire with a larger protein. Thus, our findings provide some evidence for the screening E6 epitopes in addition to E7 as targets for treatment design.

To design therapeutic vaccine with better efficacy, HLA types cannot be ignored. Among HLA serotypes, HLA-A shows the most polymorphisms. In our study, genotyping of the HLA alleles of 76 patients with CSCC showed that HLA‐A*02:01, A*03:01, and A*24:02 were the most frequent alleles. This result is consistent with the worldwide frequency distributions of HLA-A alleles among CC patients in the MHC database [30]. HLA-A*02:01 is the first noteworthy allele of the HPV-restricted epitope. Ressing ME et al. reported that E7-derived peptides have the highest affinity for binding to the HLA-A*02:01 molecule [28]. However, these common allele carriers did not show different patterns as for the multiple overlapping peptides response in our study. Instead, we found that the HLA-A*31:01 allele occurred more frequently in patients who responded to multiple overlapping E6 peptides. Yao et al. predicted 59 epitopes of HPV-16 E6, including the combination of epitopes FAFRDLCIVYR_52-62_ of E6 (HLA-A*02:06, HLA-A*31:01) [30]. Unfortunately, one of the five patients with HLA-A*31:01 died after 38 months of follow-up; thus, this allele was not associated with a better prognosis in our study. What we also noticed is that the frequency of HLA-A*02:07 was higher in patients not responding to the E6 peptide and was associated with poor prognosis in our study. Meanwhile, the CCRT rates of A*02:07 carrier group and non-carrier group were 59% (7/12) and 61% (39/64), respectively. Therefore, this conclusion was not significantly affected by chemotherapy. We have previously reported an association between A*02:07-B*46:01 haplotype and the risk of nasopharyngeal carcinoma [31]. The results of the above studies suggest HLA type can affect the antigen selection and presentation thus the intensity of T cell response. To achieve the best protection from the therapeutic vaccines exploiting T cell functions, it might be worth to find the most appropriate epitopes based on HLA type.

The results of a recent study including more than 18,700 patients with 17 types of solid tumour revealed that CD8 + cytotoxic T cells had the greatest impact on patient survival among all the immune cells [32]. The positive prognostic value of CTL epitopes is significant for drug development. CTLs express T cell receptors (TCR) on their surface. The binding of TCR and peptide–MHC class I complexes is stabilised by co-receptors. Although the epitopes for antitumor effects could be predicted using peptide-sensitized PBMCs and isolated CD8 + CTLs responses, the precise definition of CTL epitopes is important. Riemer AB et al. found that CTLs recognised and lysed target cells presenting E7_11-19_—but not E7_11-20_—peptide. However, these two epitopes are restricted to HLA-A*02:01 [33]. In our study, both E6 and E7 can elicit T cell responses in CCSC patients. The results of ELISpot experiments on E6 and E7 multiple overlapping peptides revealed some features for the combination of peptides that induce specific immune responses. These peptides are mainly located in the second half of the E6 and E7 proteins, especially E6. Therefore, additional studies of the 10-amino acid epitopes CQKPLCPEEK, EKQRHLDKKQ, and KQRFHNIRGR in E6 peptides 14–17 and HVDIRTLEDL and DLLMGTLGIV in E7 peptides 9–11 are warranted.

## Conclusion

In conclusion, HPV16 E6-specific T cell response in the peripheral blood of CSCC patients was related to lymph node metastasis. T cell response stimulated by multiple overlapping peptides was related to the prognosis. Moreover, as their immune response may be weak and their prognosis poor, CCRT and follow-up are essential for HLA*02:07 carriers. Finally, the screening of E6 epitopes showed great value as an important target of T cell immunity, which provides evidence for the development of HPV-related cancer immunotherapy.

## Supplementary Information

**Additional file 1.** Detailed positive information of HPV16 E6 single peptide-specific T cell response in each patient.

**Additional file 2.** Detailed positive information of HPV16 E7 single peptide-specific T cell response in each patient.

## Data Availability

The datasets used and/or analyzed during the current study are available from the corresponding author on reasonable request.

## Abbreviations

CEA: Carcinoembryonic antigen
CI: Confidence interval
CSCC: Cervical squamous cell carcinoma
FIGO: International Federation of Gynecology and Obstetrics
HLA: Human leukocyte antigen
HR: Hazard ratio
HR-HPV: High risk human papillomavirus
OS: Overall survival
SCC-Ag: Squamous cell carcinoma associated antigen
TSGF: Tumour specific growth factor
